# Chronic Obstructive Pulmonary Disease and Subsequent Overall and Lung Cancer Mortality in Low-Income Adults

**DOI:** 10.1371/journal.pone.0121805

**Published:** 2015-03-26

**Authors:** Melinda C. Aldrich, Heather M. Munro, Michael Mumma, Eric L. Grogan, Pierre P. Massion, Timothy S. Blackwell, William J. Blot

**Affiliations:** 1 Department of Thoracic Surgery, Vanderbilt University Medical Center, Nashville, TN, United States of America; 2 Division of Epidemiology, Department of Medicine, Vanderbilt University Medical Center, Nashville, TN, United States of America; 3 International Epidemiology Institute, Rockville, MD, United States of America; 4 Tennessee Valley Health System Veterans Affairs, Nashville, TN, United States of America; 5 Division of Pulmonary and Critical Care Medicine, Thoracic Program, Vanderbilt Ingram Cancer Center, Vanderbilt University Medical Center, Nashville, TN, United States of America; 6 Departments of Medicine, Cell and Development Biology, and Cancer Biology, Vanderbilt University Medical Center, Nashville, TN, United States of America; Clinica Universidad de Navarra, SPAIN

## Abstract

**Background:**

Chronic obstructive pulmonary disease (COPD) is a known risk factor for lung cancer and a leading cause of mortality in the U.S., but its impact may not be fully appreciated, especially among low-income populations in the southeast where COPD prevalence and lung cancer incidence are elevated.

**Methods:**

We conducted a prospective study among 26,927 low-income adults age 40–79 in the Southern Community Cohort Study who had a Center for Medicare and Medicaid Services (CMS) encounter prior to enrollment and were followed for a median of over 6 years. Using a validated algorithm for assessing COPD from CMS claims data, we estimated COPD prevalence and potential misreporting. From Cox proportional hazard models, we computed overall and lung cancer-specific mortality according to COPD status.

**Results:**

The overall prevalence of CMS-diagnosed COPD was 16%, but was twice as high among whites as blacks. Only 35% of these individuals, however, self-reported having COPD, with underreporting significantly greater for blacks than whites. Smoking-adjusted all-cause mortality was increased by 1.7-fold and lung cancer mortality by 2.3-fold among those with a CMS COPD diagnosis, with similar patterns in blacks and whites, but no excess was found among those self-reporting COPD and without CMS confirmation.

**Conclusion:**

The prevalence of COPD in this low-income population may be greater than previously recognized and misreporting is common. COPD is associated with elevated lung cancer mortality, even among those not self-reporting the condition.

## INTRODUCTION

Chronic obstructive pulmonary disease (COPD) is a well-known risk factor for lung cancer [1,2,3,4,5,6,7,8] and investigators have voiced the need for integrated research between COPD and lung cancer to understand their common epidemiology which in turn may suggest improved strategies for reducing the burden from both conditions [9]. Lung cancer is the leading cause of cancer-related mortality in the United States and COPD is the third leading cause of overall mortality, and the two combine to create a tremendous public health burden, causing substantial morbidity, disability, and mortality [10,11]. New data from the Behavioral Risk Factor Surveillance System (BRFSS) provide a 9.6% nationwide prevalence of self-reported COPD among adults over age 40 and demonstrate that COPD varies geographically across the United States, with the highest prevalence of COPD in Southern states [10]. While these data demonstrate the substantial burden of COPD, the population sampled by the BRFSS is generally of higher income than the low-income populations most afflicted by the disease [11]. Furthermore, limited data exist assessing COPD and lung cancer mortality [12], particularly among low-income individuals, and relatively few studies have examined these associations in blacks compared to whites [13,14,15].

Individuals often underreport COPD and the condition may be underdiagnosed in as many as 60–85% of patients, primarily those with mild to moderate disease [16,17,18]. Furthermore, self-reports of COPD may sometimes be inaccurate, so that the true prevalence of COPD across the United States is unknown. We report the prevalence of Centers for Medicare and Medicaid Services (CMS) confirmed, as well as self-reported physician-diagnosed COPD, in a large prospective cohort of blacks and whites enrolled across 12 southern states and followed for determination of overall and lung cancer mortality.

## MATERIALS AND METHODS

### Study Design and Population

The Southern Community Cohort Study (SCCS) is an ongoing prospective observational cohort study established to examine health disparities amongst a predominantly low-income population. From March 2002 to September 2009, 72,532 adults were enrolled into the SCCS at community health clinics, institutions providing basic health care and preventative services in medically underserved geographic areas, across a 12-state area of the Southeast (Alabama, Arkansas, Florida, Georgia, Kentucky, Louisiana, Mississippi, North Carolina, South Carolina, Tennessee, Virginia, and West Virginia). Details of the SCCS study are provided elsewhere [19,20]. In brief, participants were eligible if they were English speaking, between the ages of 40–79, and not under treatment for cancer (except for nonmelanoma skin cancer) within the prior 12 months. Among the SCCS participants, a total of 27,415 had a CMS encounter (see below) prior to enrollment into the SCCS and form the cohort evaluated for COPD and subsequent mortality. The SCCS was approved by institutional review boards at Vanderbilt University and Meharry Medical College. Written, informed consent was obtained from all study participants.

#### Baseline Characteristics and Co-Morbidities

Baseline epidemiologic data were collected during in-person computer-assisted personal interviews conducted by trained interviewers at the community health centers. Self-reported information was ascertained on demographic characteristics and exposure histories, including race/ethnicity, income, tobacco smoking history, medical history, and health insurance status. Participants were also given the 10-item Center for Epidemiologic Studies Depression Scale (CESD-10) [21]. Medical histories included assessment of self-reported prior history of physician diagnosed COPD and co-morbidities, including asthma, diabetes, heart attack/coronary artery bypass surgery and depression. Those responding yes to the question “Has a doctor ever told you that you have had, or have you ever been treated for, emphysema or chronic bronchitis?” were classified as having self-reported COPD.

#### Identification of COPD Using CMS Records

The roster of SCCS participants was linked with the CMS Research Identifiable Files from January 1, 1999 through December 31, 2008 to identify all persons who had a Medicaid or Medicare encounter prior to their entry into the SCCS. We defined the start of CMS enrollment as the minimum of either the date of their first CMS claim or the first day of the month of their 65^th^ birthday. End date for CMS follow-up was the date of SCCS enrollment. CMS coverage time is defined as time between the start of CMS enrollment and date of enrollment in the SCCS cohort. To ensure internal validity, only those SCCS participants with at least one CMS encounter prior to cohort entry were included in the analyses. Among these, 49% had only a Medicaid claim, 22% only a Medicare claim, and 29% both a Medicaid and a Medicare claim; 61% of Medicare claims occurred among persons below age 65.

COPD diagnoses prior to entry into the SCCS were defined using two previously published algorithms for identification of patients with COPD [22,23]. Using the algorithm described by Mapel *et al*., we required participants to have at least one inpatient hospitalization or emergency room encounter with a COPD diagnostic code (ICD-9 491.x, 492.x, 496) or at least two professional claims having different dates of service with a COPD diagnostic code [23]. Alternatively, participants could have had a primary discharge diagnosis code for COPD (ICD-9 491.21) throughout this same time period following algorithm 4 described by Stein *et al*. [22]. We obtained medical records for 111 lung cancer patients and searched for mention of COPD diagnosis to assess the clinical validity of the CMS-identified COPD.

#### Mortality Assessment

The cohort was followed for all-cause and lung cancer (ICD10 C33-34) mortality, with the follow up time defined as the time from SCCS enrollment until date of death, loss to follow-up, or censoring through December 31, 2011, by linkage with the National Death Index and the Social Security Administration.

### Statistical Analysis

SCCS participants with unknown self-reported COPD information (N = 488) were excluded from analyses leaving 26,927 participants. Individuals were classified according to their COPD status at entry into the SCCS in the following groups: no indication of COPD; self-report only COPD; CMS diagnosis only COPD; both self-report and CMS diagnosis of COPD. Contingency table analyses were used to compare percentages across the four groups with respect to self-reported race, gender, age, education, household income, employment status, health insurance status, self-reported prevalence (yes/no) of asthma, cardiovascular disease (history of heart attack or coronary artery bypass surgery), diabetes or depression, number of such morbidities reported (0–4), smoking status (never, former, current <10 cigarettes per day [cpd], current 10–19 cpd, current 20+ cpd), body mass index (BMI<20, 20–24, 25–29, 30–34, 35+ kg/m^2^) and summary CESD-10 score (derived by assigning 0 to 3 points for each so that a maximum value of 30 could be achieved). We employed logistic regression to calculate adjusted prevalence odds ratios (ORs) and corresponding 95% confidence intervals (95% CI) of CMS-identified COPD associated with these factors. Kaplan-Meier curves were plotted to visualize differences in crude overall survival by COPD status and were compared using the log-rank test. We estimated hazard ratios (HRs) and accompanying 95% CIs for all-cause and lung cancer mortality using Cox proportional hazard models with age as the time scale, adjusted for race, sex, income, education, BMI, number of co-morbidities, CESD-10 score, CMS coverage time, and smoking status, to evaluate whether mortality differed by COPD diagnosis and between blacks and whites. We tested for differing patterns between black and white women and men by comparing models with and without cross-product terms for sex-race-COPD status. The proportional hazards assumption was assessed by including an interaction term between COPD and time and we found hazards remained constant over time. Because individuals reporting their race as other than black/African American or white were too few for stable statistical analysis, only blacks and whites are included herein. All analyses were conducted using SAS software, version 9.3 (SAS Institute, Inc.) or R version 2.15.0. Statistical tests were two-sided and an alpha of 0.05 was used to assess statistical significance.

## RESULTS

A total of 26,927 participants (N = 7,518 whites and N = 19,409 blacks) had a CMS encounter prior to enrollment into the SCCS and had self-reported information on COPD status. Median coverage time on Medicaid or Medicare for SCCS participants prior to entry into the SCCS was 4.5 years (IQR: 3.1–6.3 years). We identified 4,213 patients (16%) with COPD diagnostic codes; only 1,463 (35%) of these individuals also self-reported COPD (Table 1). Among the 4,213 patients with COPD diagnostic codes, 67% were identified from inpatient or emergency room visits and 33% were identified from outpatient visits. No difference in the age distribution was found between individuals with inpatient versus outpatient COPD diagnoses (p-value = 0.17). The crude prevalence of CMS-identified COPD, however, was much higher among whites (26%) than blacks (12%) (*P* < 0.0001) and higher among men (19%) than women (14%) (*P* < 0.0001) Underreporting of CMS-diagnosed COPD was significantly greater in blacks than whites (73% of blacks and 57% of whites underreported, *P* < 0.0001) and males than females (69% vs 63% underreported, respectively, *P* < 0.0001), but did not differ greatly by age. Table 1 shows among the entire cohort, 3,232 (12%) self-reported having been diagnosed with COPD, with 55% (overall 7% of the participants) having had no prior CMS recording of COPD. The percentages in this latter group were nearly twice as high among women than men. In review of lung cancer patient medical records, we found 62% sensitivity and 80% positive predictive value for CMS-identified COPD.

**Table 1:** Descriptive baseline characteristics of study participants according to COPD status (N = 26,927).

	Self-reported COPD only	CMS-identified COPD only	Both self-report and CMS	No COPD
	N = 1,769	N = 2,750	N = 1,463	N = 20,945
Characteristic	N (%)	N (%)	N (%)	N (%)
Race				
Whites	592 (33.5)	1,090 (39.6)	835 (57.1)	5,001 (23.9)
Blacks	1,177 (66.5)	1,660 (60.4)	628 (42.9)	15,944 (76.1)
Sex				
Male	367 (20.8)	1,159 (42.2)	513 (35.1)	6,930 (33.1)
Female	1,402 (79.3)	1,591 (57.9)	950 (64.9)	14,015 (66.9)
Race-sex				
Black Female	938 (53.0)	914 (33.2)	399 (27.3)	10,669 (50.9)
Black Male	239 (13.5)	746 (27.1)	229 (15.7)	5,275 (25.2)
White Female	464 (26.2)	677 (24.6)	551 (37.7)	3,346 (16.0)
White Male	128 (7.2)	413 (15.0)	284 (19.4)	1,655 (7.9)
Age at cohort entry, years				
40–49	816 (46.1)	687 (25.0)	367 (25.1)	9,233 (44.1)
50–59	614 (34.7)	900 (32.7)	518 (35.4)	5,772 (27.6)
≥ 60	339 (19.2)	1,163 (42.3)	578 (39.5)	5,940 (28.4)
Highest education level, years				
< 12	735 (41.6)	1,357 (49.4)	718 (49.1)	8,197 (39.1)
12	570 (32.2)	809 (29.4)	422 (28.8)	7,090 (33.9)
≥ 12	464 (26.2)	584 (21.2)	323 (22.1)	5,655 (27.0)
Unknown	3			
Household income in last year				
< $15,000	1,356 (77.2)	2,167 (79.5)	1,160 (80.0)	15,115 (72.9)
$15,000-$25,000	291 (16.6)	405 (14.9)	201 (13.9)	3,902 (18.8)
> $25,000	109 (6.2)	154 (5.7)	89 (6.1)	1,722 (8.3)
Unknown	256			
Smoking status at cohort entry				
Current	897 (50.8)	1,364 (49.6)	818 (55.9)	8,243 (39.4)
Former	417 (23.6)	796 (29.0)	482 (33.0)	4,736 (22.6)
Never	453 (25.6)	589 (21.4)	163 (11.1)	7,944 (38.0)
Unknown	25			
Median (IQR) smoking pack-years*	22.0 (10.0–38.0)	25.0 (11.9–44.0)	34.5 (17.5–55.5)	16.0 (7.0–30.0)
Min	0	0	0	0
Max	225.0	260.0	279.5	470.0
Unknown	252			
Asthma				
Yes	662 (37.6)	960 (35.0)	767 (52.7)	2,720 (13.0)
No	1,099 (62.4)	1,784 (65.0)	688 (47.3)	18,219 (87.0)
Unknown	28			
Heart attack/bypass surgery				
Yes	198 (11.2)	548 (20.0)	322 (22.0)	1,853 (8.9)
No	1,567 (88.8)	2,196 (80.0)	1,139 (78.0)	19,070 (91.1)
Unknown	34			
Diabetes				
Yes	481 (27.3)	929 (33.8)	498 (34.0)	5,816 (27.8)
No	1,284 (72.8)	1,821 (66.2)	965 (66.0)	15,121 (72.2)
Unknown	12			
Depression				
Yes	920 (52.1)	963 (35.0)	751 (51.4)	6,330 (30.2)
No	846 (47.9)	1,785 (65.0)	710 (48.6)	14,601 (69.8)
Unknown	21			
Comorbidity count^†^				
0	382 (21.8)	717 (26.2)	190 (13.1)	8,900 (42.6)
1	681 (38.9)	996 (36.4)	504 (34.7)	8,103 (38.8)
2	523 (29.9)	709 (25.9)	489 (33.7)	3,169 (15.2)
3 or 4	164 (9.4)	314 (11.5)	268 (18.5)	723 (3.5)
Unknown	95			
Median CESD-10 Score^§^ (IQR)	12.0 (7.0–17.0)	9.0 (5.0–14.0)	11.0 (7.0–17.0)	9.0 (5.0–13.0)
Unknown	148			

*Among current and former smokers

^†^Comorbidities included asthma, cardiovascular disease, diabetes and depression

^§^Center for Epidemiologic Studies Depression Scale-10 derived score. Score calculated only for participants responding to all 10 questions.

IQR = Interquartile range.

SCCS participants with self-reported or CMS-ascertained COPD had lower education (less than 12 years) and income (<$15,000 household income) compared to individuals not having a COPD diagnosis (Table 1). Individuals with COPD were more likely to be current smokers and have higher pack-years of smoking than persons without any diagnosis of COPD. Comorbidities were prevalent among SCCS participants with COPD. Self-reported asthma was nearly three times greater among individuals with COPD compared to individuals without a CMS diagnosis or self-report of COPD. Heart attack/bypass surgery and depression were more often reported among COPD participants compared to individuals without COPD. Furthermore, COPD participants were more likely to report two or more comorbidities. Using the comprehensive CESD-10 depression score, we found participants self-reporting COPD had higher median CESD-10 scores than individuals without a diagnosis of COPD, but there was no increase for those with only a CMS-diagnosis of COPD.

In multivariate logistic regression analyses, significant associations were found between COPD prevalence and race, sex, age, education, BMI, smoking, and comorbidities (Table 2). Pronounced effects were seen for smoking, with monotonically increasing risks with amount smoked rising to an OR = 4.20, 95% CI: 3.72–4.75 for heavy current smokers vs never smokers for CMS-identified COPD. A similar odds ratio was found for the association between heavy smoking and self-reported COPD (OR = 3.26, 95% CI: 2.87–3.70). COPD prevalence was strongly associated with high comorbidity score for both CMS-identified and self-reported COPD (OR = 6.88, 95% CI: 5.95–7.95 and OR = 5.30, 95% CI: 4.54–6.17, respectively, for those with 3+ vs. 0 comorbidities). The nearly 2-fold increase in CMS-diagnosed COPD among whites compared with blacks (OR = 1.96, 95% CI: 1.82–2.12), and the 40% increase among men compared with women (OR = 1.37, 95% CI: 1.27–1.49), persisted in these adjusted analyses (Table 2). A similar increase in self-reported COPD was observed among whites compared with blacks (OR = 1.73, 95% CI: 1.59–1.88), but men had a 30% decrease in self-reported COPD compared with women (OR = 0.69, 95% CI: 0.63–0.76) (Table 2).

**Table 2:** Multivariable model of the association between participant characteristics and COPD among blacks and whites participating in the Southern Community Cohort Study (N = 26,065).

	CMS-identified COPD		Self-reported COPD	
Characteristic	OR	95% CI	OR	95% CI
Race				
Black	1.00	Referent	1.00	Referent
White	1.96	1.82–2.12	1.73	1.59–1.88
Sex				
Female	1.00	Referent	1.00	Referent
Male	1.37	1.27–1.49	0.69	0.63–0.76
Household Income ($)				
< 15,000	1.00	Referent	1.00	Referent
15,000–24,000	0.82	0.74–0.91	0.93	0.84–1.04
≥ 25,000	0.66	0.56–0.77	0.90	0.77–1.07
Education (years)				
< 12	1.21	1.11–1.32	1.14	1.03–1.25
12	1.00	Referent	1.00	Referent
> 12	0.88	0.80–0.98	0.97	0.87–1.07
Enrollment age (per year)	1.05	1.045–1.054	1.01	1.007–1.017
Smoking status				
Never smoked	1.00	Referent	1.00	Referent
Former smoker	2.26	2.03–2.51	2.15	1.91–2.41
Current smoker < 10 cpd	2.96	2.59–3.39	2.32	2.02–2.68
Current smoker 10–19 cpd	3.39	2.97–3.86	2.38	2.07–2.74
Current smoker ≥ 20 cpd	4.20	3.72–4.75	3.26	2.87–3.70
BMI (kg/m^2^)				
< 20	1.74	1.46–2.06	1.25	1.03–1.52
20–24	1.00	Referent	1.00	Referent
25–29	0.83	0.74–0.92	0.74	0.65–0.83
30–34	0.79	0.71–0.89	0.80	0.70–0.90
≥35	1.07	0.95–1.20	0.96	0.85–1.08
Comorbidity count^†^				
0	1.00	Referent	1.00	Referent
1	1.75	1.59–1.92	1.89	1.70–2.11
2	3.28	2.95–3.65	3.47	3.09–3.91
3 or 4	6.88	5.95–7.95	5.30	4.54–6.17
CESD-10 Score* (per unit change)	1.004	0.997–1.010	1.03	1.02–1.04

OR = Odds Ratio; CI = Confidence Interval; cpd = cigarettes per day; In addition to the variables in the table, model is also adjusted for coverage time on Medicare or Medicaid.

*Center for Epidemiologic Studies Depression Scale-10 derived score. Score calculated only for participants responding to all 10 questions.

^†^Number of comorbidities reported from among asthma, heart attack/coronary artery bypass surgery, diabetes and depression.

A total of 862 individuals were missing covariates resulting in 26,065 individuals.

Follow up of the cohort identified 3,936 deaths overall and 318 lung cancer deaths. All-cause mortality was 1.7-fold (HR 1.7, 95% CI 1.6–1.8) greater and lung cancer 2.3-fold greater (HR 2.3, 95% CI 1.8–3.0) for those having a CMS code for COPD. Table 3 shows that the increases among those with CMS-diagnosed COPD held regardless of self-report and that self-reported COPD alone was not significantly associated with all-cause or lung cancer mortality. We also examined whether inpatient vs outpatient COPD diagnoses were predictive of all-cause and lung cancer mortality among those with a CMS-identified COPD. An inpatient diagnosis of COPD was significantly associated with greater all-cause mortality (HR = 1.84, 95% CI: 1.58–2.13) compared to an outpatient COPD diagnosis, but was not associated with lung cancer mortality (HR = 1.38, 95% CI: 0.92–2.08).

**Table 3:** All-cause and lung cancer mortality hazard ratios among study participants according to COPD status at cohort entry (N = 26,065).

	All-cause mortality		Lung cancer mortality	
Characteristic	HR	95% CI	HR	95% CI
COPD diagnosis				
None	1.00	Referent	1.00	Referent
Self-reported only	0.89	(0.77–1.04)	1.09	(0.65–1.83)
CMS	1.69	(1.57–1.83)	2.28	(1.77–2.95)
CMS only	1.65	(1.50–1.80)	2.12	(1.59–2.82)
Self-report and CMS	1.80	(1.60–2.02)	2.66	(1.88–3.76)

HR = Hazard Ratio; CI = Confidence Interval; Models are adjusted for coverage time on Medicare or Medicaid, sex, race, income, education, BMI, smoking, CESD-10 score and comorbidity count. A total of 862 individuals were missing covariates resulting in 26,065 individuals.

**Fig 1:**
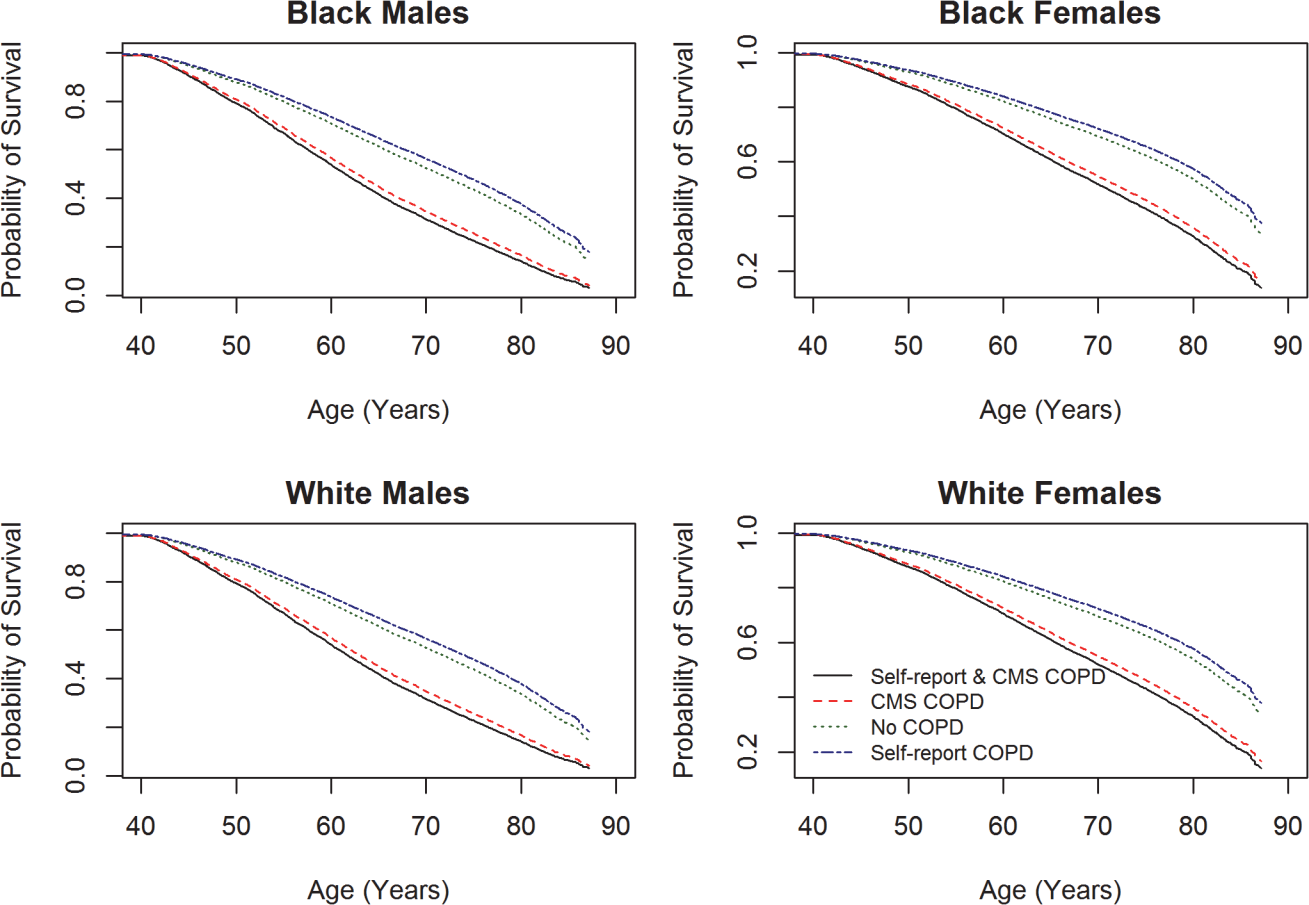
Predicted survival probabilities from Cox proportional hazard models of all-cause mortality by COPD status for each of the four race-sex groups.

Both all-cause and lung cancer mortality were significantly elevated for those with CMS-confirmed COPD across all race and sex groups (Table 4). Predicted survival probabilities from Cox proportional hazard models of all-cause mortality by COPD status for each of the four race-sex groups are shown in Fig. 1. Formal tests across the race-sex groups for differences in all-cause mortality associated with a COPD diagnosis were not statistically significant (*P* = 0.10), with no suggestion of an interaction (*P* = 0.94) for lung cancer mortality. In assessing potential risk factors for subsequent overall survival among those with CMS-diagnosed COPD, higher mortality was observed among men than women, but differences by race and socioeconomic status were not marked after adjusting for smoking status and number of comorbidities, and little differences by sex or race were seen for lung cancer (Table 5). Table 5 also shows that among SCCS participants with COPD at entry into the cohort, cigarette smoking was the dominant risk factor for subsequent lung cancer, with a greater than 10-fold excess in lung cancer mortality among those who reported smoking a pack or more per day.

**Table 4:** All-cause and lung cancer mortality hazard ratios according to COPD status at cohort entry stratified by race and sex.

		White Women (N = 4,890)		Black Women (N = 12,450)		White Men (N = 2,430)		Black Men (N = 6,295)	
Characteristic		HR	95% CI	HR	95% CI	HR	95% CI	HR	95% CI
All-cause mortality									
COPD diagnosis									
None		1.00	Referent	1.00	Referent	1.00	Referent	1.00	Referent
Self-reported		0.93	(0.67–1.30)	0.84	(0.66–1.06)	1.10	(0.74–1.65)	0.89	(0.65–1.21)
CMS		1.85	(1.48–2.30)	1.66	(1.41–1.96)	1.75	(1.40–2.18)	1.54	(1.33–1.79)
Self-report and CMS		2.17	(1.73–2.72)	1.74	(1.38–2.20)	1.64	(1.26–2.14)	1.64	(1.30–2.06)
Lung cancer mortality									
COPD diagnosis									
None	1.00		Referent	1.00	Referent	1.00	Referent	1.00	Referent
Self-reported	1.60		(0.64–4.02)	0.73	(0.26–2.03)	0.61	(0.08–4.68)	1.37	(0.55–3.44)
CMS	2.52		(1.29–4.90)	2.44	(1.37–4.35)	2.39	(1.11–5.13)	1.76	(1.10–2.82)
Self-report and CMS	2.65		(1.35–5.21)	2.74	(1.22–6.13)	2.70	(1.20–6.12)	2.81	(1.48–5.36)

HR = Hazard Ratio; CI = Confidence Interval; Models are adjusted for coverage time on Medicare or Medicaid, race, income, education, smoking, BMI, CESD-10 score, and comorbidity count. A total of 862 individuals were missing covariates resulting in 26,065 individuals.

**Table 5:** Multivariable model of risk factors for all-cause and lung cancer mortality among study participants with CMS-identified COPD (N = 4,074).

	All-cause Mortality		Lung Cancer Mortality	
Characteristic	HR	95% CI	HR	95% CI
Race				
Black	1.00	Referent	1.00	Referent
White	1.08	(0.95–1.22)	1.17	(0.81–1.69)
Sex				
Female	1.00	Referent	1.00	Referent
Male	1.47	(1.29–1.67)	1.26	(0.87–1.82)
Income ($)				
<$15,000	1.00	Referent	1.00	Referent
$15,000–24,000	0.86	(0.72–1.03)	0.97	(0.59–1.59)
≥ $25,000	0.73	(0.55–0.99)	0.37	(0.12–1.20)
Education (years)				
< 12	1.15	(1.00–1.33)	1.12	(0.75–1.68)
12	1.00	Referent	1.00	Referent
> 12	1.10	(0.92–1.32)	0.68	(0.38–1.24)
Smoking status				
Never	1.00	Referent	1.00	Referent
Former	1.37	(1.12–1.67)	4.67	(1.66–13.17)
Current, < 10 cpd	1.55	(1.21–1.97)	7.83	(2.63–23.38)
Current, 10–19 cpd	1.52	(1.20–1.93)	7.63	(2.56–22.70)
Current, 20+ cpd	1.58	(1.27–1.97)	11.42	(3.99–32.66)
BMI (kg/m^2^)				
< 20	1.28	(1.02–1.62)	1.22	(0.65–2.31)
20–24	1.00	Referent	1.00	Referent
25–29	0.81	(0.69–0.96)	0.98	(0.63–1.52)
30–34	0.70	(0.58–0.86)	0.52	(0.28–0.95)
35+	0.77	(0.64–0.93)	0.54	(0.30–0.99)
Comorbidity count				
0	1.00	Referent	1.00	Referent
1	1.26	(1.06–1.49)	1.56	(0.98–2.47)
2	1.59	(1.33–1.90)	1.57	(0.93–2.66)
3 or 4	1.46	(1.16–1.85)	1.99	(1.04–3.82)
CESD-10 Score	0.993	(0.982–1.003)	0.986	(0.956–1.017)

HR = Hazard Ratio; CI = Confidence Interval; cpd = cigarettes per day; In addition to the variables in the table, models are also adjusted for coverage time on Medicare or Medicaid.

Self-reported co-morbidities include diabetes, depression, asthma, and cardiovascular disease.

## DISCUSSION

Our investigation revealed substantial underreporting of COPD and subsequent increased lung cancer mortality among those with CMS diagnosed COPD, including among persons who did not self-report having the condition. Although several COPD studies [24,25,26,27] have examined comorbidities and overall mortality, studies jointly assessing COPD and lung cancer, especially for low socioeconomic populations or blacks, have been scant. This longitudinal multicenter study with participants age 40–79 recruited across 12 southern states found a high overall 16% prevalence of CMS-confirmed COPD among SCCS participants at cohort entry, with the prevalence approximately twice as high among whites than blacks. These individuals experienced about a 70% increase in overall mortality and 2.3-fold increase in lung cancer mortality after adjusting for smoking and other factors, confirming the major adverse health burden associated with COPD.

Approximately two-thirds of the CMS-diagnosed cases would have been missed had COPD been identified based only on participant self-report (with underreporting greater for blacks). We note these data are for a low-resource population participating in CMS, but our finding of substantial underreporting is similar to that noted in other populations [16,17,18,28], indicating poor sensitivity of self-reported COPD prevalence. Given the elevated overall and lung cancer mortality we found among those with CMS-confirmed but not self-reported COPD, there is a critical need for greater awareness and monitoring of this common disease.

The overall prevalence of self-reported COPD in the SCCS population was 12%, greater than the recently published BRFSS national average of 9.6% for adults 45 years and older, also obtained from self-reports [10], but a higher prevalence in the SCCS would be expected because of our recruitment from community health centers and the geographic location of the cohort with its high smoking prevalence and lower levels of education and income [11]. Our findings of a higher prevalence of COPD among whites compared to blacks are consistent with both national data [10] and recent COPDGene findings which found whites have more emphysema than blacks, although similar airway wall thickness and air trapping [15]. Based on CMS diagnoses, COPD prevalence was greater among men than women, whereas prevalence was higher among women than men when based on self-report. The relative increases in risk of lung cancer among those with COPD, however, were of similar magnitude in whites and blacks and men and women.

This study is subject to several limitations. The population we studied is not necessarily representative of southern residents overall or those with CMS enrollment, having lower socioeconomic status and greater co-morbidities. We did not assess pulmonary function measurements or use CT scans to evaluate emphysema. It is important to consider that ICD-9 codes reported in CMS claims are used for physician reimbursement and nuances in ICD-9 coding can complicate findings [29]. The use of ICD-9 codes to identify COPD may lack sufficient sensitivity and specificity and does not provide COPD severity. We also did not have information on medication use to control COPD. Our review of medical charts for a subset of the lung cancer patients suggested that CMS records did not detect over a third of COPD diagnoses, although positive predictive value was high. However, using examination of medical records from lung cancer cases to assess the clinical validity of CMS-identified COPD may itself have limitations. Dyspnea occurring with lung cancer could be assumed to be comorbid COPD and thus lead to an over-diagnosis of COPD in this population. Conversely, COPD may be missed in the presence of a life-threatening illness due to an inability of the individual to perform testing, and COPD diagnosed in the past, especially mild cases, may not have been recorded in the medical records pertaining to the lung cancer. Caution should be taken with interpretation of the high positive predictive value. Stein *et al*. noted that their algorithm developed for the purpose of identifying patients hospitalized for acute exacerbations of COPD had a low sensitivity, although high positive and negative predictive values suggest the algorithm has some clinical value for identifying primary acute exacerbation of COPD [22]. Our coverage time under Medicaid or Medicare was also limited so that only encounters occurring from 1999 onwards were ascertained and earlier diagnoses of COPD that were not recorded in subsequent encounters would have been missed. Coverage under Medicaid, if not continuous, could have resulted in missed diagnoses. Under-diagnosis also may have occurred since spirometry is not routinely performed in the clinic and thus clinical records may not contain COPD status. A pattern in these shortcomings is that the prevalence of COPD may be even higher than detected herein, suggesting that the public health burden of COPD and its influence upon lung cancer may be greater than previously appreciated. Although our use of electronic health records was efficient and cost-effective, refined algorithms for identifying COPD and its sequelae should continue to be developed.

Strengths of this study are numerous. One important aspect of our study is that the SCCS includes a large low-income population with a high percentage of blacks which enabled a comprehensive assessment of COPD among blacks and whites of similar socioeconomic status. Our findings extend to other U.S. studies of COPD which have primarily focused on whites and higher income populations [30,31,32] and showed that, despite a lower prevalence of COPD among blacks, lung cancer risk increased among COPD patients regardless of race. The study was conducted in a geographic catchment area where the prevalence of COPD is above the national average estimated by the BRFSS, which together with the high prevalence of smoking yielded an elevated COPD prevalence and large numbers of persons with COPD for prospective follow up. The SCCS participants were well characterized, with extensive baseline data and standardized CMS determination of medical encounters prior to cohort entry, and with linkage with the National Death Index for unbiased and complete prospective follow-up of participants for mortality outcomes. Overall, this study provides a new and important assessment of a large contingent of society at elevated risk for lung cancer mortality.

Our findings suggest that COPD is a major public health burden, with elevated overall and lung cancer mortality. The NHLBI estimates 12 million people in the United States are living with COPD, resulting in significant health care expenditures estimated to exceed $72 billion annually [33,34]. These findings underscore the need for smoking cessation and COPD screening using spirometry tests, particularly among low-income populations where smoking and COPD prevalence is high. Although there is currently no cure for COPD, early diagnosis may lead to better control of the disease, its comorbidities, and improved survival. A particularly important message from this research is that COPD is both relatively common and a strong risk factor for lung cancer. Hence lung cancer screening strategies may benefit from routine assessment of COPD status [35,36], and increased opportunities for health care providers to screen patients with COPD for lung cancer risk may lead to improved management of the condition and better clinical outcomes.
